# Supplementary material for: Tea saponin reduces the damage of Ectropis obliqua to tea crops, and exerts reduced effects on the spiders Ebrechtella tricuspidata and Evarcha albaria compared to chemical insecticides
Source: PeerJ. 2018 Mar 19;6:e4534. doi: 10.7717/peerj.4534 (PMC5863709; doi:10.7717/peerj.4534)
Supplement: Supplemental Information 1 [file peerj-06-4534-s001.docx]

**The row data of the study:**

**Table S1** **Toxicity of TS solution in 3rd-instar *Ectropis obliqua* larvae**

| Concentration of TS (mg/mL) | Total number of *E. obliqua*  larvae | Mortality of *E. obliqua*  larvae |
| --- | --- | --- |
| 300 | 30 | 22 |
|  | 30 | 20 |
|  | 30 | 18 |
| 150 | 30 | 13 |
|  | 30 | 15 |
|  | 30 | 11 |
| 75 | 30 | 10 |
|  | 30 | 8 |
|  | 30 | 9 |
| 37.5 | 30 | 8 |
|  | 30 | 9 |
|  | 30 | 8 |
| 18.5 | 30 | 5 |
|  | 30 | 3 |
|  | 30 | 4 |
| 0 | 30 | 0 |
|  | 30 | 1 |
|  | 30 | 0 |

**Table S2** **Toxicity of TS solution in *Ebrechtella tricuspidata* adults after 48 h of treatment using different reagents**

| Treatment | Concentration  (mg/mL) | Total number of  *E. tricuspidata* | Mortality of  *E. tricuspidata* |
| --- | --- | --- | --- |
| 10% Bi EC | 0.01 | 20 | 14 |
|  |  | 20 | 16 |
|  |  | 20 | 18 |
| 50% Di SC | 1.2 | 20 | 8 |
|  |  | 20 | 8 |
|  |  | 20 | 10 |
| TS | 300 | 20 | 2 |
|  |  | 20 | 4 |
|  |  | 20 | 4 |
| Control |  | 20 | 0 |
|  |  | 20 | 0 |
|  |  | 20 | 0 |

**Table S3** **Toxicity of TS solution in *Evarcha albaria* adults after 48 h of treatment using different reagents**

| Treatment | Concentration  (mg/mL) | Total number of  *E. albaria* | Mortality of  *E. albaria* |
| --- | --- | --- | --- |
| 10% Bi EC | 0.01 | 20 | 14 |
|  |  | 20 | 16 |
|  |  | 20 | 14 |
| 50% Di SC | 1.2 | 20 | 6 |
|  |  | 20 | 6 |
|  |  | 20 | 10 |
| TS | 300 | 20 | 2 |
|  |  | 20 | 4 |
|  |  | 20 | 6 |
| Control |  | 20 | 0 |
|  |  | 20 | 0 |
|  |  | 20 | 0 |

**Table S4 The controlling efficacy of 30%TS (w/v) and chemical insecticides against the *Ectropis obliqua* larvae**

| Treatment | Dose  (g a.i. ha-1) | Pre-treatment count | Post-treatment count | | | |
| --- | --- | --- | --- | --- | --- | --- |
|  |  |  | 1 d | 3 d | 5 d | 7 d |
| 10% Bi EC | 7.5 | 12 | 2 | 1 | 3 | 5 |
|  |  | 10 | 2 | 2 | 4 | 4 |
|  |  | 10 | 1 | 1 | 4 | 5 |
| 50% Di SC | 45 | 11 | 7 | 2 | 5 | 4 |
|  |  | 14 | 7 | 1 | 4 | 4 |
|  |  | 10 | 6 | 2 | 5 | 6 |
| 30% TS WG | 362.5 | 10 | 8 | 4 | 2 | 4 |
|  |  | 15 | 12 | 6 | 3 | 5 |
|  |  | 12 | 10 | 7 | 4 | 4 |
| CK |  | 10 | 9 | 9 | 11 | 8 |
|  |  | 10 | 10 | 9 | 10 | 9 |
|  |  | 12 | 12 | 11 | 13 | 11 |

**Table S5 The number and identification (families) of collected spiders in different treatment plots**

| Spider  families | Treatment | | | | | | | |
| --- | --- | --- | --- | --- | --- | --- | --- | --- |
|  | 10% Bi EC | | 50% Di SC | | 30% TS WG | | CK | |
|  | Pre-C | Post-C | Pre-C | Post-C | Pre-C | Post-C | Pre-C | Post-C |
| Salticidae | 2 | 1 | 2 | 0 | 1 | 1 | 2 | 1 |
|  | 1 | 0 | 1 | 0 | 2 | 2 | 1 | 2 |
|  | 2 | 0 | 1 | 0 | 0 | 1 | 2 | 1 |
| Thomisidae | 1 | 0 | 2 | 0 | 2 | 1 | 0 | 1 |
|  | 2 | 1 | 1 | 0 | 1 | 0 | 3 | 0 |
|  | 1 | 1 | 2 | 0 | 1 | 1 | 1 | 2 |
| Theridiidae | 1 | 1 | 0 | 1 | 0 | 1 | 1 | 1 |
|  | 2 | 0 | 1 | 0 | 1 | 1 | 1 | 1 |
|  | 1 | 0 | 1 | 1 | 1 | 1 | 2 | 2 |
| Araneidae | 0 | 0 | 0 | 0 | 1 | 0 | 0 | 0 |
|  | 1 | 1 | 1 | 0 | 0 | 1 | 0 | 0 |
|  | 0 | 0 | 1 | 0 | 1 | 0 | 0 | 0 |
| Linyphiidae | 0 | 0 | 1 | 1 | 0 | 1 | 1 | 1 |
|  | 0 | 0 | 0 | 1 | 2 | 1 | 0 | 2 |
|  | 0 | 1 | 0 | 1 | 1 | 0 | 1 | 1 |
| Agelenidae | 0 | 0 | 0 | 0 | 1 | 0 | 0 | 0 |
|  | 0 | 0 | 1 | 0 | 0 | 0 | 0 | 0 |
|  | 1 | 0 | 0 | 0 | 0 | 1 | 0 | 0 |
| Tetragnathidae | 0 | 0 | 0 | 0 | 1 | 0 | 1 | 0 |
|  | 0 | 0 | 0 | 0 | 0 | 0 | 0 | 1 |
|  | 0 | 0 | 0 | 0 | 0 | 1 | 0 | 0 |
| Gnaphosidae | 1 | 1 | 0 | 0 | 0 | 0 | 0 | 0 |
|  | 0 | 1 | 0 | 0 | 0 | 0 | 1 | 0 |
|  | 0 | 0 | 0 | 0 | 0 | 0 | 0 | 0 |
| Oxypidae | 1 | 0 | 0 | 0 | 0 | 0 | 0 | 1 |
|  | 0 | 0 | 0 | 0 | 1 | 0 | 0 | 1 |
|  | 1 | 0 | 1 | 0 | 0 | 0 | 0 | 1 |
| Clubionidae | 0 | 0 | 0 | 0 | 0 | 0 | 0 | 0 |
|  | 1 | 0 | 0 | 0 | 0 | 0 | 0 | 0 |
|  | 0 | 0 | 1 | 0 | 0 | 0 | 1 | 0 |

Pre-C, Pre-treatment count; Post-C, Post-treatment count (7 d).

Spiders were identified from the reference keys and catalogues provided by Yin et al. (2012) and the World Spider Catalog.

**Table S6** **The effects of 30% (w/v) TS on GST activity in 3rd-instar *Ectropis obliqua* larvae at different times**

| Treatment | The activities of GST at different times (U/mg) | | | | |
| --- | --- | --- | --- | --- | --- |
|  | 6 | 12 | 24 | 48 | 96 |
| TS | 0.8042 | 0.8815 | 0.7759 | 0.4656 | 0.3615 |
|  | 0.7965 | 0.8479 | 0.7591 | 0.4199 | 0.3144 |
|  | 0.8137 | 0.8831 | 0.7867 | 0.4597 | 0.3395 |
|  | 0.7828 | 0.8471 | 0.7559 | 0.4472 | 0.3378 |
| CK | 0.4597 | 0.4663 | 0.4739 | 0.4818 | 0.4557 |
|  | 0.4779 | 0.4487 | 0.4493 | 0.4604 | 0.4718 |
|  | 0.4882 | 0.4701 | 0.4902 | 0.5003 | 0.4669 |
|  | 0.5118 | 0.4529 | 0.4570 | 0.4733 | 0.4580 |

**Table S7 The effects of 30% TS (w/v) on CES activity in 3rd-instar *Ectropis obliqua* larvae at different times**

| Treatment | The activities of CES at different times (U/mg) | | | | |
| --- | --- | --- | --- | --- | --- |
|  | 6 | 12 | 24 | 48 | 96 |
| TS | 0.0597 | 0.0569 | 0.0499 | 0.0502 | 0.0502 |
|  | 0.0605 | 0.0531 | 0.0522 | 0.0511 | 0.0466 |
|  | 0.0629 | 0.0577 | 0.0485 | 0.0479 | 0.0449 |
|  | 0.0702 | 0.0555 | 0.0522 | 0.0488 | 0.0479 |
| CK | 0.0799 | 0.0769 | 0.0807 | 0.0792 | 0.0647 |
|  | 0.0817 | 0.0807 | 0.0819 | 0.0777 | 0.0599 |
|  | 0.0788 | 0.0794 | 0.0843 | 0.0758 | 0.0616 |
|  | 0.08 | 0.075 | 0.0827 | 0.0765 | 0.0638 |

**Table S8 The effects of 30% TS (w/v) on AChE activity in 3rd-instar *Ectropis obliqua* larvae at different times**

| Treatment | The activities of AChE at different times (U/mg) | | | | |
| --- | --- | --- | --- | --- | --- |
|  | 6 | 12 | 24 | 48 | 96 |
| TS | 0.0633 | 0.0661 | 0.0579 | 0.0594 | 0.0589 |
|  | 0.0609 | 0.0639 | 0.0586 | 0.0538 | 0.0593 |
|  | 0.0641 | 0.0647 | 0.0607 | 0.0587 | 0.0611 |
|  | 0.0629 | 0.0681 | 0.0628 | 0.0585 | 0.0615 |
| CK | 0.0825 | 0.1102 | 0.0855 | 0.0733 | 0.0713 |
|  | 0.0817 | 0.1134 | 0.0861 | 0.0729 | 0.0685 |
|  | 0.0778 | 0.0886 | 0.0810 | 0.0688 | 0.0702 |
|  | 0.0816 | 0.0938 | 0.0842 | 0.0706 | 0.0688 |

**Table S9 The effects of 30% TS (w/v) on POD activity in 3rd-instar *Ectropis obliqua* larvae at different times**

| Treatment | The activities of POD at different times (U/mg) | | | | |
| --- | --- | --- | --- | --- | --- |
|  | 6 | 12 | 24 | 48 | 96 |
| TS | 9.2873 | 9.4582 | 9.5102 | 10.0082 | 9.5587 |
|  | 9.0105 | 9.0973 | 9.4928 | 10.1217 | 9.7206 |
|  | 8.9976 | 9.4435 | 9.3997 | 9.9965 | 9.6964 |
|  | 9.1490 | 9.5974 | 9.4909 | 9.9720 | 9.7031 |
| CK | 7.7792 | 7.7565 | 9.9233 | 10.3167 | 9.5164 |
|  | 7.6933 | 8.2074 | 9.8024 | 9.9906 | 9.3815 |
|  | 7.4899 | 7.9931 | 9.9215 | 9.8452 | 9.4907 |
|  | 7.6104 | 7.7334 | 9.760 | 9.7619 | 9.4674 |

**Fig. S1 The photograph of Wang Dazhen tea plantation**

**
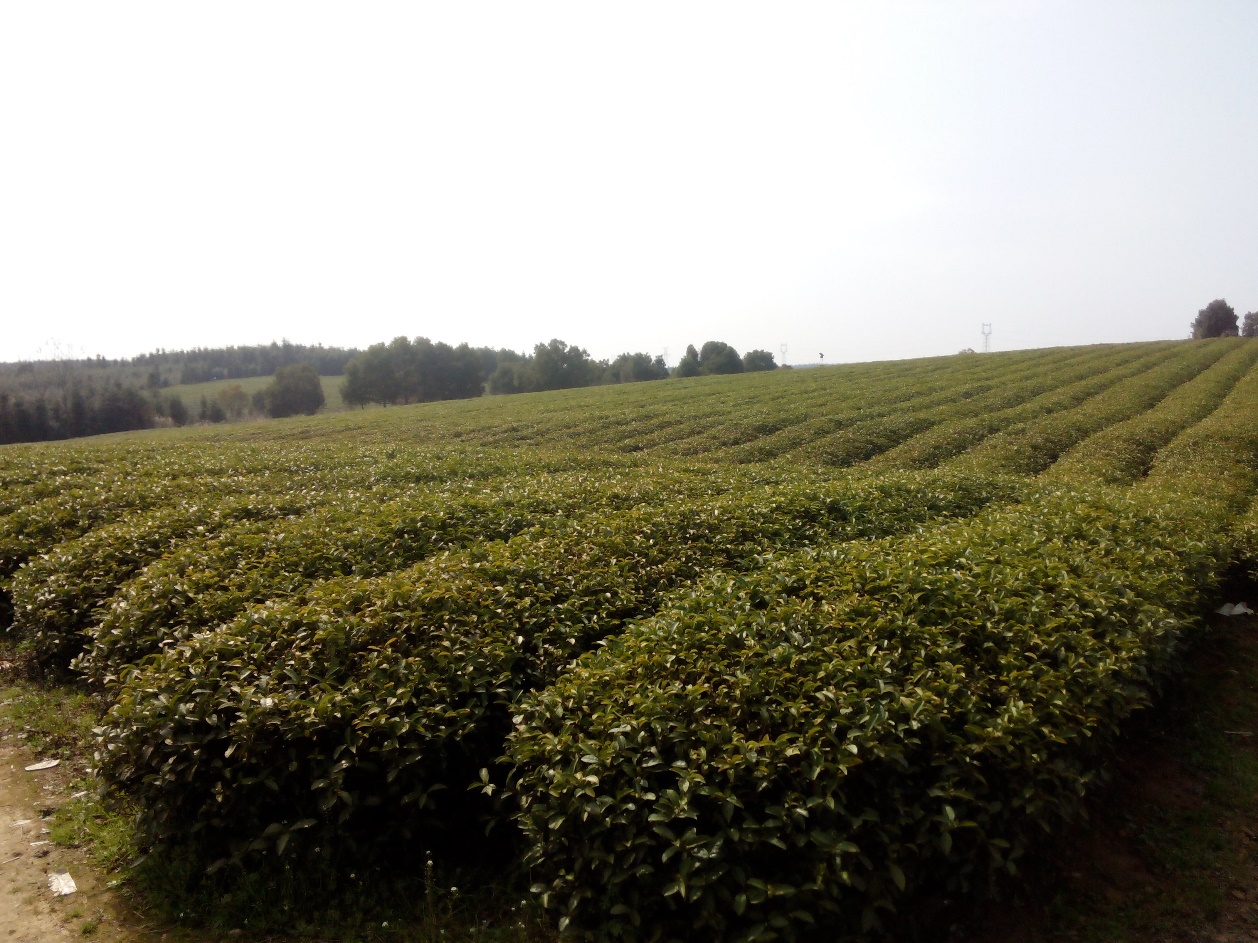
**

The total area of the collection site is about 6.5 ha with parallel rows of tea plants about 100 m long and 1 m apart. Each tea bush is 80 cm length × 80 cm width (Photograph credit: Chi Zeng).

**Supplementary references**

World Spider Catalog, Natural History Museum Bern. Available at <http://wsc.nmbe.ch/> (accessed on 5 February 2018).

Yin CM, Peng XJ, Yan HM, Bao YH, Xu X, Tang G. 2012. *Fauna Hunan: Araneae in Hunan, China*. Changsha: Hunan Science and Technology Press.
